# Maternal–Fetal Implications of Chikungunya Virus Infection: An Updated Review

**DOI:** 10.3390/diagnostics15222843

**Published:** 2025-11-10

**Authors:** Luisa Scomparim, Gustavo Yano Callado, Livian Cássia De Melo, Marina Macruz Rugna, Stefany Silva Pereira, Liris Naomi Noguchi, Camilla Martins dos Santos Maia, Evelyn Traina, Geraldo Duarte, Antonio Braga, Edward Araujo Júnior

**Affiliations:** 1 Discipline of Woman Health, Municipal University of São Caetano do Sul (USCS), São Caetano do Sul 09521-160, SP, Brazil; luisa.brito@uscsonline.com.br (L.S.); livian.melo@uscsonline.com.br (L.C.D.M.); marina.rugna@uscsonline.com.br (M.M.R.); stefany.pereira@uscsonline.com.br (S.S.P.); 2 Discipline of Woman Health, Albert Einstein Israelite College of Health Sciences, Albert Einstein Israelite Hospital, São Paulo 05652-900, SP, Brazil; gycallado@gmail.com; 3 Discipline of Woman Health, Santo Amaro University (UNISA), São Paulo 04829-300, SP, Brazil; lirisnaomin@gmail.com; 4 Department of Obstetrics, Paulista School of Medicine, Federal University of São Paulo (EPM-UNIFESP), São Paulo 04023-062, SP, Brazil; camilla.maia@unifesp.br (C.M.d.S.M.); evelyntraina@gmail.com (E.T.); 5 Department of Gynecology and Obstetrics, Ribeirão Preto School of Medicine, University of São Paulo (FMRP-USP), Ribeirão Preto 14049-900, SP, Brazil; gduarte@fmrp.usp.br; 6 Department of Gynecology and Obstetrics, School of Medicine, Federal University of Rio de Janeiro (UFRJ), Rio de Janeiro 22240-003, RJ, Brazil; bragamed@yahoo.com.br; 7 Department of General and Specialized Surgery, School of Medicine and Surgery, Federal University of the State of Rio de Janeiro (UNIRIO), Rio de Janeiro 20271-062, RJ, Brazil; 8 Postgraduate Program in Applied Health Sciences, University of Vassouras, Vassouras 27700-000, RJ, Brazil

**Keywords:** chikungunya, vertical transmission, congenital infection, epidemiological surveillance, maternal–fetal health

## Abstract

Chikungunya virus (CHIKV) infection during pregnancy represents an increasing public health concern, especially in endemic and epidemic regions. The main concern is vertical transmission, particularly during the peripartum period, which can lead to severe neonatal outcomes such as encephalopathy, hematologic abnormalities, and long-term neurodevelopmental impairment. This review synthesizes current knowledge on pathophysiology, clinical manifestations, diagnosis, maternal and neonatal outcomes, and management of CHIKV infection in pregnancy. Diagnosis relies on clinical evaluation supported by laboratory confirmation, RT-PCR in the acute phase and IgM serology thereafter. Treatment is supportive, using acetaminophen as first-line therapy and corticosteroids for selected refractory cases. No antivirals or vaccines are approved for use in pregnancy as of 2025. Prevention is centered on vector control, personal protection, and epidemiological surveillance. Delivery planning and neonatal monitoring are essential when infection occurs close to term due to the high risk of peripartum transmission. Despite growing recognition of CHIKV’s maternal–fetal impact, significant gaps remain regarding long-term outcomes and optimal management strategies. Strengthening prenatal care, neonatal preparedness, and surveillance systems is crucial to mitigate adverse outcomes and inform future clinical and public health policies.

## 1. Introduction

Chikungunya virus (CHIKV) is an arbovirus belonging to the Togaviridae family, genus Alphavirus, first isolated in 1952 during an outbreak in the Makonde region on the border between Tanzania and Mozambique. The term “chikungunya” is derived from the Makonde language and refers to the stooped posture observed in affected individuals, a consequence of the intense polyarthralgia that characterizes the clinical picture of the disease. Transmission occurs predominantly through the bite of female mosquitoes of the *Aedes genus*, especially *Aedes aegypti* and *Aedes albopictus*, which are also vectors of other significant arboviral infections, such as Dengue, Zika, and yellow fever [1].

Following an incubation period ranging from 2 to 7 days, CHIKV infection typically presents with sudden-onset fever, polyarthralgia, myalgia, headache, maculopapular rash, and asthenia. Arthralgia may persist for weeks or even months, particularly in adults, and represents one of the most disabling features of the disease. Chikungunya infection is usually self-limited, characterized by fever and arthralgia. However, severe outcomes may occur in vulnerable populations such as the elderly, immunocompromised individuals, pregnant individuals, and neonates. In these groups, an exacerbated inflammatory response, impaired viral replication control, and physiological fragility may lead to more severe neurological, hepatic, hematological, and cardiovascular manifestations [1].

Intrauterine CHIKV infection can result in a broad spectrum of neonatal clinical presentations, ranging from nonspecific symptoms such as fever and irritability to severe conditions with potential risk of death. Reported complications in the literature include neonatal encephalopathy, seizures, hematological disorders, hepatitis with significant elevation of liver enzymes, hepatosplenomegaly, as well as respiratory and cardiovascular manifestations [2,3]. Delayed neurological and behavioral consequences have also been documented, with evidence of cognitive, motor, and psychosocial impairments in children exposed to CHIKV in utero, even in the absence of identifiable morphological abnormalities at birth [1,4,5].

Although fetal infection is considered rare, vertical transmission in viremic mothers can reach up to 50% of newborns. This risk becomes particularly relevant during large epidemics in countries with high birth rates, where up to half of the cases may progress to severe forms [6]. Unlike the Zika virus, whose congenital transmission is frequently associated with infections in early pregnancy, CHIKV is more commonly transmitted from mother to fetus in acute infections occurring in the final weeks of gestation, with clinical manifestations observed during the first week of life [2,6]. Children exposed to CHIKV during the first and second trimesters of pregnancy tend to be protected against maternal–fetal transmission in the perinatal period as well as against postnatal transmission, since maternal IgG antibodies with neutralizing activity are transferred to the fetus in utero [6].

Most infections are mild and resolve without specific treatment, but close monitoring is recommended near delivery due to potential neonatal exposure. The continued geographic expansion of CHIKV, driven by urbanization, climate change, and vector proliferation, underscores the need for surveillance and targeted prenatal management in endemic and epidemic regions to prevent neonatal complications.

This review provides an updated synthesis incorporating the epidemiological evolution of CHIKV in Brazil and Latin America up to 2024, with emphasis on data from the 2023–2024 outbreaks and the first neurodevelopmental follow-up cohort from Brazil. It also integrates recent developments in vaccine research, including the IXCHIQ vaccine (a live attenuated vaccine approved by the US FDA in 2023 for ≥18 years, which represents a milestone in the prevention of CHIKV, although it is not licensed for use in pregnancy or childhood after 2025), and practical guidance for prenatal surveillance in endemic regions, which were not addressed in previous systematic reviews.

## 2. Methods

This narrative review was conducted through a structured literature search in PubMed, Scopus, SciELO, and LILACS databases covering the period January 2010 to August 2025. The search combined the terms “chikungunya”, “pregnancy”, “vertical transmission”, “maternal infection”, and “neonatal outcomes”. Reference lists of retrieved articles and key guidelines from WHO and the Brazilian Ministry of Health were also reviewed. Studies included if they reported clinical, epidemiological, diagnostic, or management data related to CHIKV infection in pregnancy. Both original research and review articles were considered, without restrictions on language.

## 3. Epidemiology

CHIKV infection in pregnancy follows the global geographic distribution of the virus, which occurs predominantly in tropical and subtropical regions, with sustained transmission in the Americas, Africa, Asia, and the islands of the Indian and Pacific Oceans. Since the virus was introduced into the Americas in 2013, it has spread rapidly, with large-scale outbreaks in Caribbean, Central, and South American countries, including Brazil, Colombia, Paraguay, the Dominican Republic, Haiti, Martinique, Guadeloupe, and Puerto Rico. The risk of infection is highest during epidemic periods, often associated with the rainy season, when *Aedes aegypti* proliferation increases 2013 [7,8,9,10,11,12]. Regional disparities in nutrition, sanitation, and vector control policies likely influence reported outcomes and case detection rates. Such environmental and social determinants may partly explain the observed heterogeneity between studies from the Caribbean, Brazil, and Indian Ocean regions. Furthermore, surveillance data likely underestimate the true incidence due to asymptomatic or misclassified cases and variable diagnostic capacity across regions.

The number of reported cases worldwide is substantial, outbreaks have been reported in several nations, and the virus currently has endemic circulation in approximately 100 countries, with more than 5 million accumulated cases [7]. In 2023, the Americas reported over 120,000 cases of CHIKV with 82 confirmed deaths associated with the disease, resulting in an incidence rate of 67.4 cases per 100,000 inhabitants [7]. The outbreak began in East Africa, reached Réunion Island in 2005, peaking in 2006, and has since triggered several epidemics throughout the Indian Ocean region [13]. The first outbreak of CHIKV infection in Brazil was documented between 2013 and 2014, involving 37 individuals, mostly from Haiti and the Dominican Republic. Between January 2016 and July 2021, 733,703 cases of CHIKV were reported in Brazil [5].

In Brazil, between 2016 and 2018, 3262 pregnant individuals with confirmed CHIKV infection gave birth to live neonates, with most cases reported in the Northeast (65.2%) and Southeast (15.8%) regions. The highest incidence occurred in the states of Ceará (28.3%), Pernambuco (17.8%), and Rio de Janeiro (11.9%). Approximately 40% of infections occurred during the second trimester, and the mean maternal age was ~26 years, with 53.4% of women aged 20–29 years. Most pregnant individuals were of mixed race (62.5%) and unmarried (45.3%), with 8–11 years of education (65.8%). Comorbidities such as diabetes (0.89%) and hypertension (1.81%) were infrequent. Maternal comorbidities (e.g., chronic hypertension, pregestational diabetes, and anemia) were uncommon in included cohorts and inconsistently ascertained; reported hypertension or diabetes were typically <2% in recent Brazilian surveillance datasets, limiting between-study adjustment. Because comorbidity prevalence tracks maternal age and socioeconomic status, failure to account for these factors may bias crude associations between CHIKV exposure and adverse perinatal outcomes. Regarding mode of delivery, 45.1% had vaginal births and 54.9% underwent cesarean section. The most common symptoms were fever (79.6%), myalgia (64.6%), headache (63.0%), and arthritis (24.5%). A large proportion lived in urban areas (85%). Among the 3332 confirmed maternal cases, only four maternal deaths were recorded (0.12%), one during pregnancy (second trimester) and three in the postpartum period. None of the deaths occurred near the time of delivery or required hospitalization. Among the newborns, 50.6% were male and 49.4% female; 86.2% were born at term (37–41 weeks), 77.1% were of mixed race, 5.9% had low birth weight, and 1.0% presented with malformations (33 cases). A total of 39 malformations were reported, most of which occurred in pregnancies affected during the second trimester, with polydactyly and Down syndrome being the most common. Maternal diagnosis was associated with a protective effect against low birth weight [14].

Pregnant individuals experiencing socioeconomic vulnerability, such as those in Grenada (low income, lack of window screens, increased exposure), showed a higher risk of infection [15]. Pregnancy has been reported as a factor associated with acute hospitalization [16]; however, this finding is largely based on data from [2], where the observed increase was restricted to cases presenting symptoms of CHIKV. Thus, it should not be interpreted as a generalized higher risk of hospitalization among pregnant individuals. Additional hospital-based data, such as a study in Colombia, documented 60 pregnant individuals hospitalized with confirmed CHIKV infection, 15 of whom were in the acute phase during delivery. Although some presented with severe cases, no maternal deaths were reported [15].

Since the 2005–2006 outbreak in Réunion Island, it has been established that acute maternal viremia at delivery can lead to severe neonatal manifestations, including neurological and systemic involvement. The severity follows a U-shaped pattern: higher in neonates and the elderly, and milder in young, healthy adults [17]. Therefore, maternal CHIKV infection is prevalent in tropical and endemic areas, with a significant impact on vulnerable populations. Targeted obstetric surveillance and post-infection follow-up for both mothers and neonates are essential [13].

## 4. Transmission

CHIKV is primarily transmitted through the bite of infected *Aedes* mosquitoes, particularly *Aedes aegypti* and *Aedes albopictus*. Prior to urban spread, CHIKV circulates in nature through a rural enzootic cycle, which constitutes the main viral maintenance mechanism. In this cycle, the virus is maintained among non-human primates and wild mosquitoes, both acting as natural reservoirs in forested or savannah areas [18].

The increasing proximity between wild and human environments—often resulting from deforestation, urban expansion, or anthropogenic activities—facilitates the spillover of the virus into urban settings. In these contexts, the human–mosquito–human transmission cycle is established, in which mosquitoes acquire the virus from infected individuals during viremia [18]. Following an extrinsic incubation period ranging from 2 to 10 days, the mosquito becomes capable of transmitting the virus through saliva when biting other susceptible individuals, contributing to the emergence of periodic outbreaks. A key feature of vector-borne transmission is that only female *Aedes* mosquitoes are hematophagous and, therefore, capable of transmitting CHIKV. This behavior is driven by the need for blood-derived proteins essential for egg development, which leads females to feed on human blood. During this process, the mosquito’s saliva may contain viral particles capable of infecting the host [19].

In addition to vector transmission, CHIKV can be vertically transmitted, particularly in the perinatal period. This form of transmission occurs due to the virus’s ability to cross the placental barrier, disrupting placental homeostasis through maternal–fetal blood exchange, especially during labor. Evidence demonstrates the presence of the virus in trophoblastic, decidual, Hofbauer, and fetal endothelial cells, confirming CHIKV’s placental tropism [20,21].

Vertical transmission can occur at any stage of pregnancy and has been associated with adverse outcomes such as spontaneous abortion and inflammatory placental lesions, especially when infection occurs during the first or second trimester. However, the highest transmission rates are observed in the perinatal period, when maternal viremia coincides with the days preceding delivery, with vertical transmission rates reaching up to 50% [20,21,22,23]. Reported vertical transmission rates range widely (5–50%) depending on study design, diagnostic methods, and timing of maternal viremia. The highest rates are observed when infection occurs within five days before delivery. Such wide variation largely reflects heterogeneity in study design, diagnostic criteria, and timing of maternal infection, limiting comparability between studies.

Regarding other potential routes of transmission, although CHIKV RNA has been detected in biological fluids such as breast milk and genital secretions, there is no robust evidence supporting transmission via sexual contact or breastfeeding. There are also isolated reports of percutaneous transmission, such as through needlestick injuries in healthcare settings or laboratory exposure. However, to date, no confirmed cases of transmission via blood transfusion have been documented in humans, and this route remains only a theoretical risk [24,25].

## 5. Clinical Presentation

Although clinical presentation in pregnant individuals is similar to that of the general population, physiological and immunological changes inherent to pregnancy can alter symptom expression, inflammatory responses, and maternal–fetal risks. Yet, most available studies are retrospective or hospital-based, which may overrepresent symptomatic or severe cases while excluding community infections. Table 1 summarizes the main clinical manifestations of CHIKV infection during pregnancy and their respective pathophysiological mechanisms.

Given the epidemiological significance and the morbidity and mortality associated with the disease, it is important to understand the underlying pathophysiology of each clinical symptom.

### 5.1. High-Grade Sudden-Onset Fever

Fever is typically the earliest manifestation, with temperatures frequently exceeding 39 °C. It results from activation of the innate immune system and release of pyrogenic cytokines such as IL-1β, IL-6, and TNF-α, which act on the hypothalamus. During pregnancy, the febrile response may be attenuated; however, maternal fever in the first trimester is associated with an increased risk of miscarriage and congenital anomalies, particularly when sustained [23,28,29].

### 5.2. Polyarthralgia and Inflammatory Arthritis

Symmetrical, intense, and debilitating joint pain is one of the most prominent complaints during maternal infection. It results from the virus’s tropism for synovial tissues, intra-articular replication, and recruitment of inflammatory cells (macrophages, CD4+ T lymphocytes, neutrophils). The release of IL-6, TNF-α, and IL-17 contributes to chronic synovial inflammation. In pregnant individuals, joint pain may impair mobility, sleep, and well-being, potentially affect prenatal care and labor, and increase the risk of chronic disease progression [27,28,29].

### 5.3. Pruritic Maculopapular Rash

The cutaneous rash, commonly appearing between the 2nd and 5th day of infection, is attributed to local vasodilation and dermal infiltration by lymphocytes and immune complexes. During pregnancy, it may be confused with pregnancy-specific dermatoses such as gestational pruritus. However, its centrifugal distribution (trunk and limbs) and association with fever help differentiate it [28,29].

### 5.4. Peripheral Edema

Edema of the hands, feet, and ankles occurs due to increased vascular permeability mediated by cytokines such as TNF-α, IL-1, and bradykinin. While edema is common in physiological pregnancy, the presence of painful, asymmetric, and sudden-onset swelling suggests an inflammatory viral component and must be distinguished from obstetric causes such as preeclampsia [28].

### 5.5. Myalgia and Profound Fatigue

Generalized muscle pain, particularly in the lower limbs, is mediated by local muscular inflammation, myocyte injury, and nociceptor sensitization. Persistent fatigue, which may last for weeks, is related to sustained pro-inflammatory states and mitochondrial dysfunction. In pregnant individuals, these symptoms can compromise nutritional status, adherence to prenatal care, and functional capacity [29].

### 5.6. Neurological Manifestations

Pregnant individuals may develop neurological complications such as encephalitis, Guillain–Barré syndrome, and myelitis, particularly in the third trimester. These conditions are associated with molecular mimicry, autoimmune activation, and possibly direct invasion of the central nervous system (CNS). Pregnancy, being an immunomodulated state (Th2 predominance), may exacerbate autoimmune responses or hinder control of viral replication within the CNS [28,30].

### 5.7. Hemorrhagic Manifestations and Obstetric Complications

Although rare, there are reports of mucocutaneous and postpartum bleeding in pregnant individuals with CHIKV infection, particularly in the context of associated thrombocytopenia. The infection may increase the risk of preterm birth, oligohydramnios, fetal distress, and emergency cesarean delivery, especially when infection occurs late in pregnancy. Where reported, anemia, chronic hypertension, and pregestational diabetes should be modeled as potential confounders; however, few studies provide adjusted estimates, precluding firm causal inference. We therefore interpret risk signals cautiously and recommend multivariable models that include maternal comorbidities and trimester of infection, with sensitivity analyses excluding high-risk pregnancies. In intrapartum infections, there is a high risk of vertical transmission, often associated with severe neonatal outcomes [31]. The inconsistency likely arises from differences in case definitions, timing of infection confirmation, and small sample sizes that limit statistical power.

### 5.8. Vertical Transmission and Neonatal Consequences

Risk was concentrated near delivery: infections in the third trimester—particularly peripartum—carried the highest transmission probability (reports up to ~50%) and the most severe neonatal presentations, while early-gestation infections showed low or unquantified transmission risk. The virus can cross the placental barrier during maternal viremia. Infected neonates typically exhibit symptoms between the 3rd and 7th day of life, including fever, rash, irritability, thrombocytopenia, encephalopathy, seizures, and in severe cases, death. Follow-up durations and neurodevelopmental assessment tools vary widely, complicating interpretation of long-term sequelae across cohorts. Histopathological findings include chronic villitis, deciduitis, and detection of viral RNA in the placenta [6,23,32].

### 5.9. Immunological Aspects of Pregnancy

Pregnancy is characterized by a predominantly Th2 immune response, which supports fetal tolerance but impairs the control of intracellular viruses such as CHIKV, which require an effective Th1-mediated response. Additionally, altered T cell and NK cell function during pregnancy may contribute to higher viremia and exacerbated clinical symptoms in infected pregnant individuals [6,23,26,31].

### 5.10. Maternal–Fetal Outcomes

**Figure 1:**
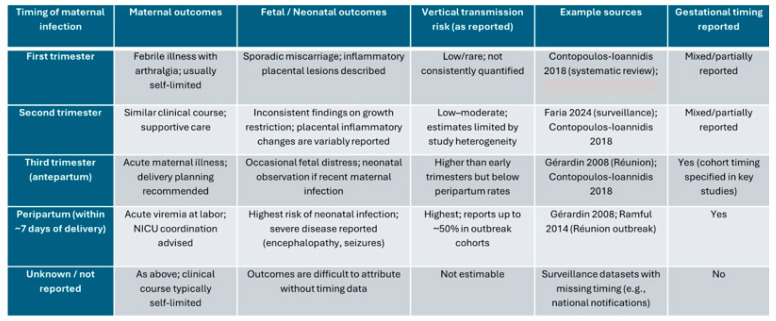
Outcomes stratified by trimester of maternal chikungunya virus infection. Abbreviations: NICU, neonatal intensive care unit [4,5,7,27]. “As reported” reflects study authors’ timing and outcome definitions.

Adverse outcomes associated with chikungunya virus infection depend largely on the gestational stage at which infection occurs. To address gestational age as a confounder, outcomes were stratified by trimester of maternal infection. Early infections (first/second trimester) were primarily associated with sporadic miscarriage and inflammatory placental lesions, whereas late infections (third trimester) prompted enhanced surveillance and delivery planning given potential neonatal exposure. However, comparison across studies is hindered by methodological differences, incomplete timing data, and variable case definitions. The available evidence is summarized in Figure 1.

## 6. Diagnosis

The diagnosis of chikungunya is primarily clinical, as symptoms such as acute-onset fever and arthralgia have a high positive predictive value in endemic areas. Additionally, nonspecific laboratory findings such as lymphopenia, thrombocytopenia, hypocalcemia, and elevated serum levels of aspartate aminotransferase and alanine aminotransferase support the presence of viremia [16]. However, confirmatory diagnostic testing is required due to the clinical overlap with other arboviruses, particularly Zika and Dengue, as proper case management depends on accurate identification of the infecting agent.

During the acute phase of infection, the preferred diagnostic method is viral RNA detection via reverse transcription polymerase chain reaction (RT-PCR) performed on a serum sample. This should be collected during the first week of illness, ideally within the first six or seven days after symptom onset, as viremia is typically high during this period and RT-PCR demonstrates excellent sensitivity and specificity [16,33,34]. After day six of illness, viremia declines, and serological testing for antibodies, especially IgM, becomes more appropriate. Enzyme-linked immunosorbent assay (ELISA) or indirect immunofluorescence assays can detect IgM antibodies, which begin to rise during this stage and may remain detectable for weeks to months. The sensitivity of these tests exceeds 90% when performed after day seven of symptom onset. Additionally, detection of IgG antibodies may indicate convalescence or past infection [34,35,36]. False-positive IgM results can occasionally occur due to cross-reactivity with dengue and Zika viruses and other alphaviruses (e.g., Mayaro virus, O’Nyong-Nyong virus) or nonspecific antibodies. In such cases, plaque reduction neutralization tests (PRNT) can be employed to confirm infection, given their high specificity [34,37].

Imaging studies are not diagnostic tools per se but are indicated for the assessment of potential maternal and fetal complications. In particular, in cases of perinatal fetal involvement, neuroimaging—especially magnetic resonance imaging (MRI)—is essential for detailed evaluation of the central nervous system. Radiological findings may vary according to the stage of infection. During the acute phase, diffusion-restricted areas may be observed in the subcortical white matter of both cerebral hemispheres and in the corpus callosum, with a perivascular distribution pattern. In subacute or late phases, bilateral cystic lesions may predominate, which may or may not be accompanied by cerebral atrophy [38]. Thus, imaging findings are not sufficiently specific to confirm acute chikungunya infection, and serological tests remain essential for laboratory confirmation. Furthermore, longitudinal follow-up is critical to monitor and detect early neurological or systemic complications related to maternal–fetal infection.

Taken together, these considerations highlight the importance of tailoring diagnostic testing to the phase of maternal infection and suspected fetal involvement. Table 2 summarizes the recommended approach.

## 7. Treatment

Management of CHIKV infection during pregnancy is fundamentally symptomatic, as no specific antiviral therapies or licensed vaccines are currently available for clinical use [7,39,40,41]. Recommended treatment includes rest, adequate hydration, and the use of analgesics and antipyretics—preferably paracetamol (acetaminophen)—for fever and joint pain control. This recommendation is especially important in dengue-endemic areas due to the increased risk of hemorrhagic complications associated with non-steroidal anti-inflammatory drugs (NSAIDs) in potential dengue coinfections [7,42].

NSAIDs may be considered, particularly if the patient has been afebrile for at least 48 h and arthritic symptoms persist [7,42]. In cases of persistent or difficult-to-manage joint pain, systemic corticosteroids such as prednisone may be cautiously used, with individualized assessment due to the inherent risks of corticosteroid therapy during pregnancy [42]. In the context of chronic infection, defined by persistent arthralgia, management follows principles similar to those used in rheumatologic conditions and may include physical therapy, NSAIDs, corticosteroids, and, in selected cases, disease-modifying antirheumatic drugs (DMARDs) such as methotrexate, sulfasalazine, and hydroxychloroquine. However, the evidence supporting their use is limited, based on small clinical trials with a high risk of bias and no consensus regarding their safety or efficacy during pregnancy. The use of immunosuppressive agents and biologics remains experimental and is not routinely recommended [43]. In summary, management of CHIKV infection during pregnancy remains largely supportive, focusing on symptom control and maternal comfort. In this context, the use of corticosteroids or DMARDs in pregnancy should be considered expert opinion rather than evidence-based practice. Methotrexate is contraindicated during pregnancy (Food and Drug Administration Category X—studies in pregnant individuals have demonstrated fetal risk or abnormalities, and the potential risks clearly outweigh any possible benefits), while hydroxychloroquine and sulfasalazine may be used cautiously when benefits outweigh risks.

For the fetus in utero, no proven interventions are currently available to prevent or treat chikungunya virus infection. Vertical transmission occurs primarily when maternal viremia coincides with the peripartum period. No antivirals have been approved for maternal or fetal use, and management remains supportive [7,40,41]. Experimental studies in animal models suggest that human convalescent immunoglobulin may have prophylactic or therapeutic potential for exposed neonates, but this approach is not yet available for clinical use [44]. In clinical practice, pregnant individuals with confirmed infection should undergo serial ultrasound evaluations every 4 weeks, or every 2 weeks when approaching term or if complications are suspected, to assess fetal growth and amniotic fluid. Doppler studies are indicated in cases of suspected fetal distress, and delivery is recommended in tertiary centers equipped with neonatal intensive care facilities when maternal infection occurs within 7 days before delivery.

**Figure 2:**
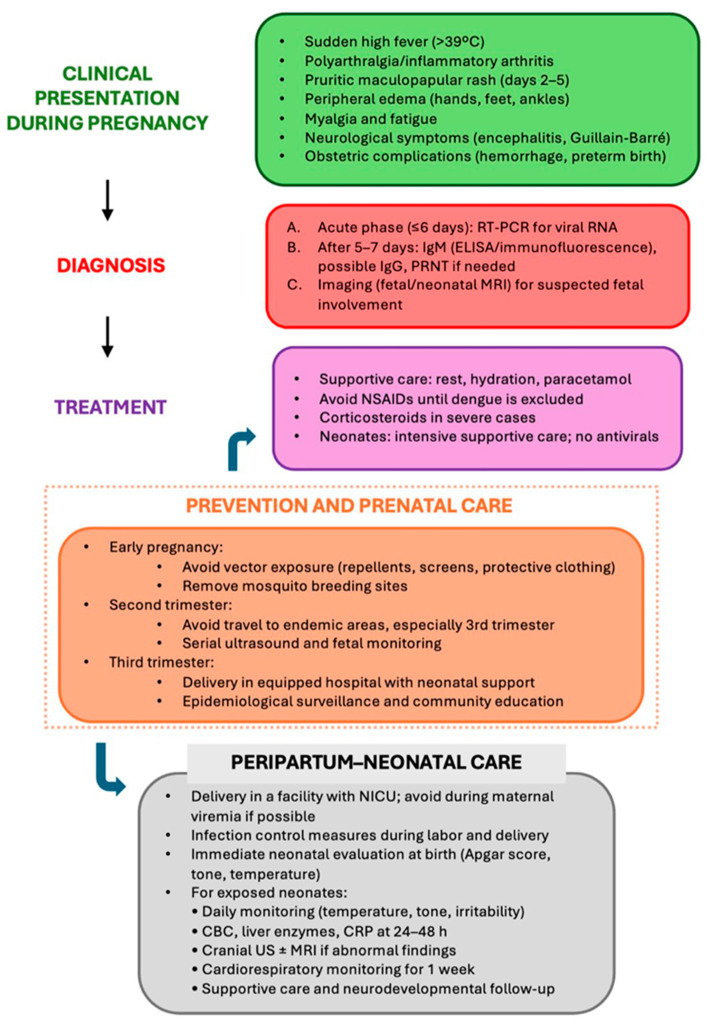
Clinical management flowchart of chikungunya virus infection during pregnancy.

After birth, neonates exposed to acute maternal infection should undergo close monitoring for signs of disease, including severe neurological and systemic manifestations (Figure 2). Treatment is supportive, with intensive monitoring, fluid management, temperature control, and ventilatory or hemodynamic support as needed [7,44]. No antiviral therapies or immunotherapies are approved for neonatal use [7,40,44].

In summary, management of CHIKV infection in pregnant individuals and neonates relies on supportive care, including judicious use of analgesics and anti-inflammatory agents. NSAIDs should be avoided until dengue has been ruled out. Corticosteroids may be used in selected cases. No specific antiviral or immunotherapeutic treatments are currently approved, and experimental interventions such as convalescent immunoglobulins are not yet part of standard clinical practice [7,40,42,43,44].

## 8. Prevention

The prevention of intrauterine CHIKV infection is primarily based on reducing the risk of maternal infection during pregnancy, as vertical transmission occurs predominantly in the perinatal period and is associated with potentially severe neonatal disease, including neurological manifestations and increased morbidity and mortality risk [45,46]. The main preventive strategies include:

1. Avoiding vector exposure: Individual protection measures are recommended, such as wearing long-sleeved clothing, using insect repellents deemed safe for pregnancy, installing window and door screens, and sleeping under mosquito nets—especially during the day, when vectors are most active. Elimination of mosquito breeding sites, such as standing water containers, is essential at both household and community levels [45];
2. Vaccination: The IXCHIQ vaccine, recently approved by the FDA for adults ≥18 years, is not yet licensed for use in pregnant individuals or children. Therefore, vaccination is not currently a viable preventive strategy during pregnancy, although it may be considered for household contacts and at-risk professionals depending on future guidelines and availability [46];
3. Avoiding travel to endemic areas: Pregnant individuals, especially in the third trimester, should be advised against traveling to regions with active CHIKV transmission due to the increased risk of maternal infection and its potential neonatal consequences [45];
4. Community-based vector control: Collective measures such as public health education campaigns, can help reduce the incidence of infection in the general population, indirectly protecting pregnant individuals [47,48];
5. Passive immunoprophylaxis: Experimental studies indicate that administration of anti-CHIKV immunoglobulin may represent a promising prophylactic strategy for neonates exposed during delivery. However, this approach remains investigational and is not currently implemented in clinical practice [44];
6. Epidemiological surveillance and case tracking: Implementing epidemiological surveillance measures in endemic areas enables early identification of cases in pregnant individuals, allowing targeted clinical interventions and appropriate neonatal monitoring [4].

## 9. Prenatal Care

Prenatal care plays a key role in early detection, monitoring, and appropriate management of infections that may compromise fetal health, including CHIKV. Given the virus’s potential for vertical transmission—particularly when infection occurs in the third trimester—obstetric follow-up must be diligent to ensure identification and monitoring of at-risk pregnancies [49].

Prenatal visits should include guidance on preventing infection, emphasizing the elimination of *Aedes aegypti* breeding grounds, the proper use of insect repellents, and other personal protection measures to reduce maternal exposure to the vector, such as wearing light-colored clothing that covers as much skin as possible [49]. For infected pregnant individuals, there is a high demand for maternal and fetal health monitoring, including serial ultrasound evaluations to detect early fetal abnormalities, assess growth, and evaluate fetal well-being. Monitoring maternal viremia, particularly in acute infections near term, is crucial for assessing the risk of vertical transmission [3,50].

In this context, delivery should take place in a hospital equipped with neonatal intensive care units (NICUs) and immediate multidisciplinary support. Trained healthcare teams must be present, with the capacity to promptly identify and manage complications related to congenital infection. Furthermore, in anticipation of adverse fetal outcomes, such as preterm birth, corticosteroid therapy and magnesium sulfate administration are indicated. In patients with renal impairment, magnesium sulfate requires dose adjustment and monitoring [4,48].

Thus, integrating epidemiological surveillance with high-quality prenatal care is essential for mitigating the adverse effects of intrauterine CHIKV infection, helping to reduce neonatal morbidity and mortality and improve maternal and perinatal outcomes [3] (Figure 2).

Neonates born to mothers with CHIKV infection near delivery require close observation because viremia at the time of labor markedly increases the risk of perinatal transmission. Early recognition of neurological or systemic manifestations is essential to prevent complications. 

## 10. Conclusions

Intrauterine infection with CHIKV poses a significant emerging threat to maternal–fetal health, particularly in endemic contexts or during epidemic outbreaks [4,6]. While most infections in pregnant individuals are self-limited, vertical transmission—especially when maternal viremia occurs in the peripartum period—can lead to severe neonatal outcomes, including encephalopathy, hematologic abnormalities, and impaired neuropsychomotor development [4,6].

The complex pathophysiology of the infection, combined with the unique immunological profile of pregnancy, makes this population especially vulnerable. Appropriate management requires rigorous prenatal surveillance, effective vector control measures, and hospital infrastructure capable of providing specialized neonatal support [6].

Despite recent advances in understanding congenital CHIKV infection, important gaps remain regarding mechanisms of vertical transmission and the long-term neurodevelopmental impact of intrauterine exposure. Future studies should apply standardized definitions and prospective designs to support meta-analytic synthesis and guide evidence-based policy. By integrating 2023–2024 Latin American epidemiology, emerging IXCHIQ vaccine considerations, and new neurodevelopmental data, this review provides a clinically actionable synthesis that informs trimester-specific care, peripartum preparedness, and public health strategies aimed at mitigating adverse maternal and neonatal outcomes.

## Figures and Tables

**Table 1:** Summary of studies on Chikungunya Virus (CHIKV) infection during pregnancy with clinical presentations and perinatal implications.

Author/Year	Country	Sample Size	Main Findings	Level of Evidence
Contopoulos-Ioannidis et al., 2018 [4]	Multi-country review	242 pregnancies	12% preterm birth; 2% vertical transmission	Systematic review
Faria et al., 2024 [7]	Brazil	1530 cases	Increased incidence during 2023–2024 outbreak; no maternal deaths	Cohort
Quintans et al., 2025 [26]	Brazil	68 infants	Neurodevelopmental delay in 9% of exposed infants	Cohort
Gérardin et al., 2008 [27]	Réunion Island	600 pregnancies	48% peripartum infections linked to neonatal encephalopathy	Cohort
Ramful et al., 2014 [5]	Réunion Island	7 cases	Severe neonatal CHIKV requiring NICU support	Case report
Summary of clinical presentation				
Acute Chikungunya Infection During Pregnancy	– Sudden-onset high fever – Severe, symmetric polyarthralgia with joint swelling – Myalgia and prolonged fatigue – Pruritic maculopapular rash – Swelling of hands and feet – Headache, photophobia – Nausea, vomiting, persistent arthritis – Rarely: neurological involvement (encephalitis, Guillain-Barré syndrome), hemorrhagic manifestations, or myocarditis			
Vertical Transmission and Congenital Infection (Newborn)	– Fever, rash, neonatal irritability – Thrombocytopenia, anemia, hepatosplenomegaly – Seizures, encephalopathy, brain lesions (calcifications, ventriculomegaly) – Retinopathy and cataract – Neuromotor abnormalities and delayed neurodevelopment – High neonatal morbidity and mortality in severe cases			
Obstetric Complications	– Increased risk of preterm birth – Oligohydramnios – Acute fetal distress – Emergency cesarean section – Vertical transmission particularly if maternal infection occurs within 5 days before delivery			

**Table 2:** Diagnostic approach for chikungunya virus infection according to clinical scenario.

Clinical Scenario	Optimal Test	Remarks
<7 days since symptom onset	RT-PCR on serum	Highest sensitivity; differentiates from Zika and Dengue viruses
>7 days	IgM or ELISA	May persist for weeks; beware of cross-reactivity
Indeterminate or discordant results	PRNT reference laboratory	Confirms true infection
Suspected fetal involvement	Ultrasound ± MRI	Assess the central nervous system and hepatic findings

Abbreviations: RT-PCR, reverse-transcription polymerase chain reaction; IgM, immunoglobulin M; ELISA, enzyme-linked immunosorbent assay; PRNT, plaque reduction neutralization test; MRI, magnetic resonance imaging.

## Data Availability

The original contributions presented in this study are included in the article. Further inquiries can be directed to the corresponding author.
